# Process evaluation of health system costing – Experience from CHSI study in India

**DOI:** 10.1371/journal.pone.0232873

**Published:** 2020-05-13

**Authors:** Shankar Prinja, Sehr Brar, Maninder Pal Singh, Kavitha Rajsekhar, Oshima Sachin, Jyotsna Naik, Malkeet Singh, Himanshi Tomar, CHSI Study Collaborating Investigators, Pankaj Bahuguna, Lorna Guinness

**Affiliations:** 1 Department of Community Medicine and School of Public Health, Post Graduate Institute of Medical Education and Research, Chandigarh, India; 2 Department of Health Research, Ministry of Health and Family Welfare, Government of India, New Delhi, India; 3 Independent Researcher, Imperial College London, London, England; Murdoch University, AUSTRALIA

## Abstract

**Background:**

A national study, ‘Costing of healthcare services in India’ (CHSI) aimed at generating reliable healthcare cost estimates for health technology assessment and price-setting is being undertaken in India. CHSI sampled 52 public and 40 private hospitals in 13 states and used a mixed micro-costing approach. This paper aims to outline the process, challenges and critical lessons of cost data collection to feed methodological and quality improvement of data collection.

**Methods:**

An exploratory survey with 3 components–an online semi-structured questionnaire, group discussion and review of monitoring data, was conducted amongst CHSI data collection teams. There were qualitative and quantitative components. Difficulty in obtaining individual data was rated on a Likert scale.

**Results:**

Mean time taken to complete cost data collection in one department/speciality was 7.86(±0.51) months, majority of which was spent on data entry and data issues resolution. Data collection was most difficult for determination of equipment usage (mean difficulty score 6.59±0.52), consumables prices (6.09±0.58), equipment price(6.05±0.72), and furniture price(5.64±0.68). Human resources, drugs & consumables contributed to 78% of total cost and 31% of data collection time. However, furniture, overheads and equipment consumed 51% of time contributing only 9% of total cost. Seeking multiple permissions, absence of electronic records, multiple sources of data were key challenges causing delays.

**Conclusions:**

Micro-costing is time and resource intensive. Addressing key issues prior to data collection would ease the process of data collection, improve quality of estimates and aid priority setting. Electronic health records and availability of national cost data base would facilitate conducting costing studies.

## Introduction

India’s three-tier healthcare system serves a population of over 1.3 billion across 28 states and 9 union territories, aimed at providing affordable and accessible primary healthcare. The three levels are primary (sub-centre and primary health centre-PHC), secondary (Community health centre-CHC) and tertiary (district and speciality hospitals). Since its inception, the Indian healthcare system has strived towards universal health coverage and this is being realized with the Ayushman Bharat scheme. It has two components, Health and Wellness Centres (HWCs) for strengthening of primary healthcare services and Ayushman Bharat—Pradhan Mantri Jan Aarogya Yojana (AB-PMJAY), a tax funded health insurance scheme. AB-PMJAY aims to provide a coverage of INR 500000 to over 100 million vulnerable families for secondary and tertiary care services at public and private facilities. [1] Owing to the lack of scientific evidence, the reimbursement rates of AB-PMJAY packages had been defined by a consultative process.

The Government of India has also established a health technology assessment agency called Health Technology Assessment India (HTAIn) to strengthen evidence-based policy making. [2] HTA requires cost data inputs including health system costs and out of pocket expenditure (OOP). Health system cost entails the cost borne by the provider or health system for service delivery while OOP is the financial cost borne by patients in accessing healthcare services. Significant data exists for OOP through various household surveys, such as various rounds of household survey by National Sample Survey Organization (NSSO). However, there is limited information on health system cost since the systems for compiling and collecting cost data are relatively under-developed. [3,4]

Addressing the information gap, studies are now being undertaken more frequently to estimate health system costs in Indian settings. Of the existing cost studies, some are procedure/surgery specific [5–11], disease/treatments specific [12–20] and some estimate public healthcare facility costs [21–27]. The existing costing studies largely focus on a single health facility or condition; do not cover all health care services and do not cover all types of health care providers, most importantly the private sector. Hence, they fail to capture the likely heterogeneity in costs across different types of providers and settings. Further, these studies have been conducted using different methodologies and perspectives, in different regions and at different levels of healthcare. As such they lack generalizability and cannot be used in national policy making. To support HTAIn’s efforts in economic evaluation and to aid price-setting of the AB-PMJAY packages, a national level study titled ‘Costing of health services in India’(CHSI) was initiated by Department of Health Research (DHR), Ministry of Health & Family Welfare (MoHFW), New Delhi, in joint collaboration with Post Graduate Institute of Medical Education and Research (PGIMER) Chandigarh and respective Multi-Disciplinary Research Units (MRU) in the 13 states. The aim of the CHSI study is to generate reliable standardised cost estimates for both policy decisions and health system research at a national level and for these cost data to be used in cost-effectiveness analyses for HTA, inform price negotiations for AB-PMJAY as well as budgeting.

Economic costing of health services involves a number of sequential steps. Starting with identifying the input resources, estimating resource use, valuing resources in monetary terms and determining the service outputs. [28] The number of steps and complexities of cost data collection make it a daunting task where there is no routine cost data. Hence it is essential to determine and outline the best way to undertake this complex task, especially in multi-layered health systems. Challenges in data collection have been reported by previous costing studies both in India and elsewhere. [28,29]. These include wide variations in health care delivery infrastructure in India, non-availability of disaggregated data and hospital management information systems (HMIS). Other challenges include obtaining stock-related data, price information and in particular the continued record keeping in physical forms.

Documenting these issues will be critical for shaping cost data collection and improving data quality in the future. Despite the common knowledge around these issues, a comprehensive analysis of challenges faced during health system cost data collection across different states has not be undertaken in Indian settings. This paper reports the findings of the process evaluation of the cost data collection in the CHSI study. It outlines the process followed and challenges faced during data collection for generating national level economic costs, and identifies critical lessons which can feed into subsequent methodological improvement, as well as improve quality of data collection in future studies.

## Methodology

### Costing of Health Services in India (CHSI) study

The aim of the CHSI study was to generate cost estimates to inform price setting of AB-PMJAY packages and HTA. The study covers 13 Indian states, collecting data from 1 tertiary public hospital, 3 public district hospitals and 3–4 private hospitals in each state. A multi-stage sampling was done to represent heterogeneity based on geography, economic status, health indicators and health service utilization. The cost centres included in the study were outpatient department (OPD), inpatient department (IPD), operating theatre (OT), intensive care unit (ICU) and ancillary services. The study was conducted using an economic perspective and a mixed methodology (top-down and bottom-up). Cost data collection was led by state level team based at each MRU. A central team trained and supported the state teams, collated the state level data and provided quality assurance. Overall analysis was carried out by the central team. Ethical approval for the CHSI study was obtained from the Institutional Ethics Committee of Post Graduate Institute of Medical Education and Research Chandigarh.

### Data collection

The present process evaluation was exploratory in nature and used multiple methods. (S1 Data) Three approaches were used for data collection, namely an online survey using a semi-structured questionnaire, group discussion and the review of monitoring data. Data was collected with the aim of understanding the process of cost data collection including: time taken, resources used, challenges and solutions. It was important to take into account that in a costing activity, different kinds of information are required to be collected under each type of input resource. For example, for human resources cost estimation requires information on salary, leave, incentives and time allocation. Similarly, information on quantity, price and utility is required for consumables. Collecting each kind of cost data poses different challenges requiring varied amounts of time and resources. Therefore, in addition to assessing the overall process of data collection, the study aimed to explore differences between input resources. This study was first of its kind, hence tools were developed for data collection and validated in a national consultation with DHR and MRUs. The tools were piloted in 2 sites before initiating data collection in all 11 sites.

#### Online survey

A semi-structured tool was designed to elicit information on the data collection process and challenges faced in undertaking cost data collection. The tool had both quantitative and qualitative parameters and comprised of five sections. The first section focused on the basic details of the MRU including location, service capacity, allocated departments and time spent on data collection. The second section focused on inputs or pre-requisites for data collection and associated challenges like institutional permissions, staff recruitment and training. This section contained objective questions to understand the process followed for permissions and subjective questions to understand critical challenging areas. The third section focused on aspects of data collection specific to each input resource or output data. Respondents were required to provide detailed information on the process of data collection for each input resource such as building, human resource, equipment etc. There were 3 parts of the third section. The first part contained multiple choice questions on cost data collection process adopted. The second part comprised of a detailed table to be filled for each resource. (Table 1) The data collection team were required to rate the level of difficulty in cost data collection on a Likert scale of 1 to 10, where 1 implied no difficulty in data collection and 10 implied it was impossible to collect data. The third part captured additional data required for costing of OT procedures.

**Table 1:** Details on process of cost data collection (section 3 of questionnaire).

Information required	Description
**Time taken to complete data collection**	Details of time spent on negotiation and waiting for data and the actual time of extraction of that particular data.
**Data Source–Level of aggregation**	Details of the level at which this data was available, institution, department or the specific cost center (ICU/OPD/OT/IPD).
**Data Source–Type of data source**	Details of the form of the data–Electronic/ physical registers/conversation with personnel etc.
**Person contacted**	Details of person contacted and person who was found to be most suitable for obtaining the data.
**Personnel collecting data**	Details of the person who collected the data–Investigator/Co-Principal Investigator/Admin staff etc.
**Challenges faced**	A detail of any challenges that were faced during the collection or retrieval of the data.
**Innovative ideas used to tackle the problem**	Details of methods used to tackle the challenge and obtain the data.
**Rating of the difficulty of data collection**	The level of challenge was rated on a scale of 1 to 10 where 1 implied no difficulty and 10 implied it was not possible to collect the data. This was a subjective rating based on the experience of each site.

The final two sections of the tool elicited field team experience regarding the training and supervisory support under the CHSI study and suggestions for improvement of data collection. The survey participants included MRUs of 11 states (Andhra Pradesh, Bihar, Delhi, Gujarat, Jammu & Kashmir, Maharashtra, Odisha, Rajasthan, Tamil Nadu, Uttar Pradesh and West Bengal) involved in the CHSI study.

#### Group discussion

An open-ended group discussion was conducted after the online survey in order to illicit detailed information on key areas identified. The participants included key personnel involved in the CHSI study (state co-investigators, field staff, central supervisory and data analysis team). The key themes were multiplicity of permissions required; lack of electronic records; identification of the right data source; adapting to changing data requirements and evolvement of data collection tool.

#### Review of central monitoring data and key stakeholder interviews

The progress of data collection at field sites was closely monitored by the central (PGIMER) team in terms of time taken for data collection, number of communications required for clarifications, data pendency etc. This data was used in order to infer the time spent on data collection at each site. Secondly, in-depth interviews of central team members were conducted to capture their experiences with a special focus on quality assurance. Findings are reported in conjunction with the findings of the online survey, since core issues that came out of the survey were further elicited from these sources.

### Data analysis

#### Quantitative data

The quantitative data from the survey was collated and analysed using MS Excel 2017, for descriptive statistics. Summary statistics for questions with discrete variable responses (categorical responses such as yes and no) were summarized in the form of percentages and represented as bar graphs. Summary statistics for questions with continuous variable responses with single response were summarized as mean, median, range and standard error. These are represented as box and whisker plots and in summary tables. Summary statistics for questions with continuous variable responses with multiple responses (such as proportions of sources of price data) were summarized as median and were represented as stacked column graphs. Time was analysed in two ways, firstly the overall time lapse in cost data collection and secondly person hours actually spent in data collection activities. Further, the time spent on data collection of each input resource was compared with its share in the total cost.

#### Qualitative data

Qualitative data was obtained from three sources, namely the group discussion, interviews and the online survey. The qualitative data obtained from the group discussion was categorized thematically to delineate key challenges and best practices in data collection. The responses obtained from the group discussion were clubbed under the outlined themes and reported accordingly. The interviews of central team members were focussed on eliciting information on quality assurance and were analysed under one theme. The subjective responses to the online tool were analysed by identifying and comparing the most common themes, across the different sites.

## Results

The results are presented according to the key themes identified.

### Staff profile and training

There were 65 staff working on the costing study at the MRU level across the sites. On an average there were 5 staff members per MRU (range: 3 to 8), including 3 field officers, 1 administrative assistant guided by 1 co-principal investigator at each MRU. In addition, at some sites regular hospital staff (residents & professors) aided the data collection. Amongst the CHSI staff, 49.23% were centrally trained comprising principal investigators, professors and residents(53.13%) and field data collection staff (46.87%). The remaining staff (50.77%), primarily field investigators and administrative assistants, was trained at the MRU level by centrally trained staff. Amongst the staff, 73.3% were postgraduates and the rest were graduates.

### Time horizon of cost data collection

**Fig 1:**
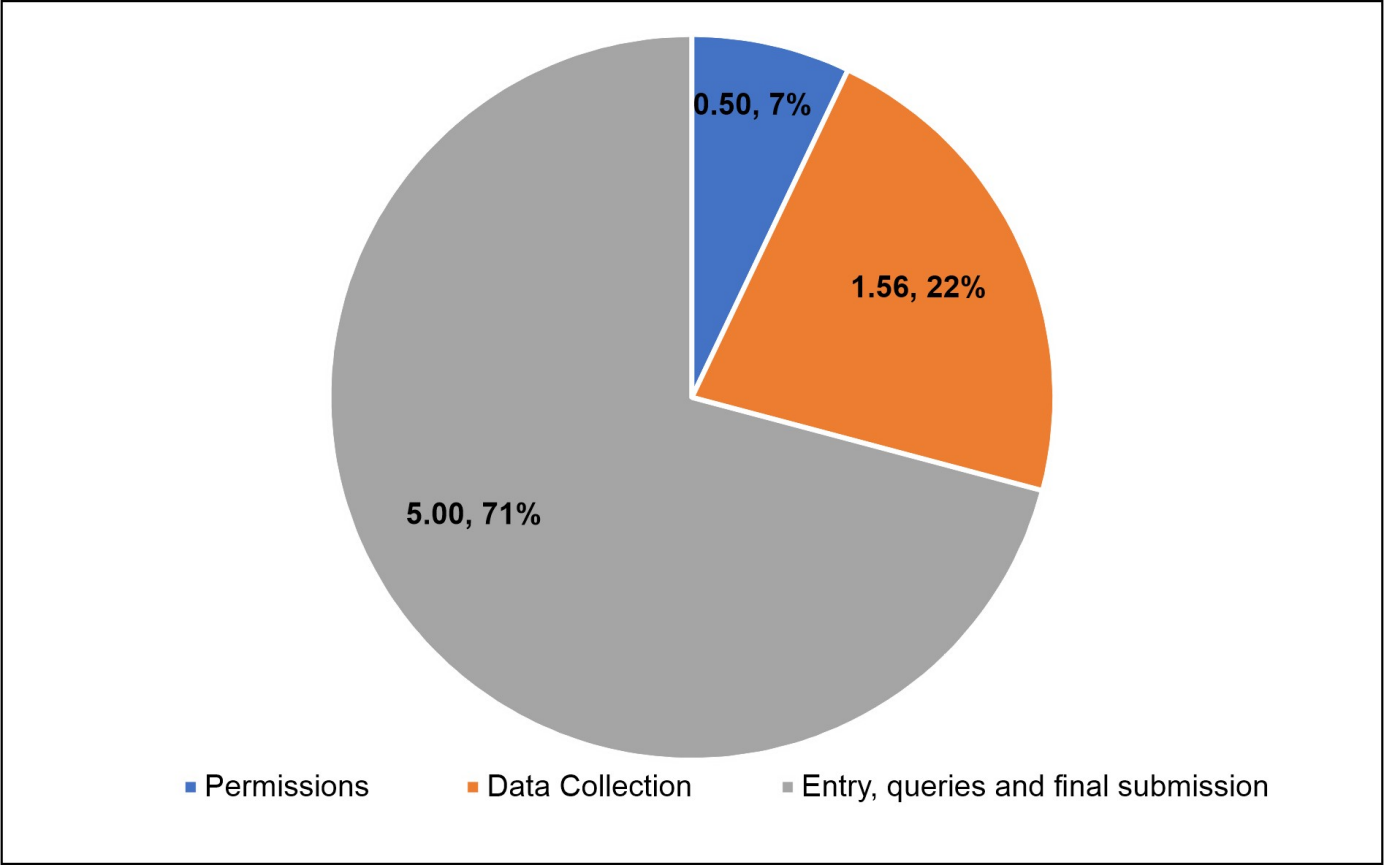
Median time of data collection (time in months).

The data collection process was considered under 3 activities; obtaining permissions, actual data collection and entry, queries & final submission which took 0.5 months(Interquartile Range(IQR) 0.28–1.5), 1.56 months (IQR 1.08–3.88) and 5 months (IQR 3.44–5.50) respectively. (Fig 1) On an average, data collection in one department of one facility covering all its services required 355 person-days.

The duration of data collection was disaggregated by the input resource level. (Table 2) Across all input resources, the time spent on negotiation and waiting was more than the actual time spent on data collection. It was found to be the longest for building and consumables, and shortest for overheads.

**Table 2:** Time required for data collection (days) per input resource.

Input resource	Data type	Time required for collection	
		Negotiation& waiting (Days) (Median, Interquartile Range)	Actual Collection (Person-days) (Median, Interquartile Range)
**Human resource**	HR Salary & Incentives	12.5 (8.13–30)	12 (5–18)
	Leave	9.75 (4.75–28.75)	8 (5–11)
	Time allocation	7 (4.88–10)	12 (9–26)
**Physical area/ building**	Building area measurement	14.50 (3.25–27.5)	17 (5–30)
	Determination of rental price	2 (1–7)	3 (1–6)
**Consumables**	Consumables used	13.75 (4.25–23.25)	15 (6–27)
	Prices of consumables	13 (6.25–26)	15 (5–25)
**Furniture / Non-consumables**	Furniture items used	6.5 (3.25–14.75)	28 (10–30)
	Prices of non-consumable items	8 (5–19.38)	15 (6–25)
	Information on average life of furniture items	4 (2.13–7)	12 (4–16)
**Equipment**	Equipment used	7 (5.50–11.63)	16 (9–26)
	Equipment procurement prices	8.50 (4.25–22.5)	15 (8–25)
	Average life of equipment	4.50 (2–7)	12 (5–16)
	Usage of equipment in different procedures	6 (4–9.25)	15 (9–18)
**Overheads**	Electricity	3 (2.25–8.88)	6 (3–14)
	Building Maintenance	3 (2.50–7)	4 (2–8)
	Equipment Maintenance	3 (2–7)	4 (2–10)
	Laundry	6 (2–21)	8 (5–10)
	Dietetics	7.25 (3.50–20.13)	8 (6–9)
	Biomedical waste management	5 (2.50–11.25)	4 (2–10)
**Service provision data**	Annual patient load data (OPD/IPD/ Surgeries)	7.25 (6–14)	20 (12–25)
	Average time of each procedure	5.50(2–13)	4 (3–13)
	Average length of stay in ICU and IPD	4.50 (2–13.75)	4 (2–11)
	Diagnostics used in each procedure	3.50 (2–7)	6 (4–15)
	Average OPD visits (pre and post procedure)	2 (2–7)	6 (3–11)
	List of drugs and consumables purchased by patient before procedure	2 (1–7)	4 (2–6)

**Fig 2:**
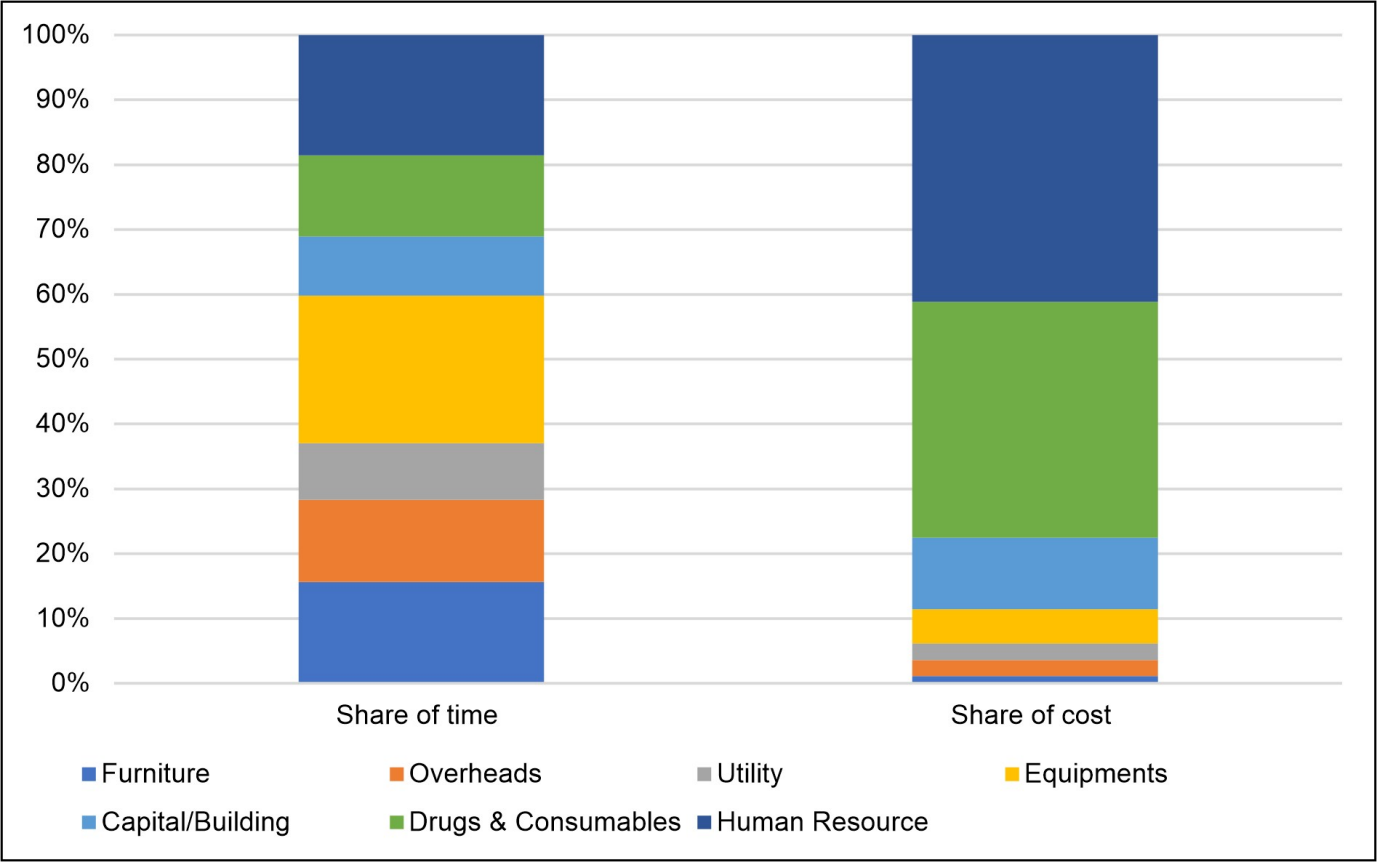
Share of input resources in data collection time and cost.

Fig 2 compares the proportion of time taken to collect data on each input resource with its share in the total cost. It was observed that while human resources and drugs & consumables contributed to 78% of the total cost, consuming only 31% of data collection time. However data collection of Furniture, Overheads and Equipment consumed 51% of the total time contributing to only 9% of the total cost.

### Permission for cost data collection

In most MRUs (9/11) the prime person to be contacted for data collection permission was the Dean/Administrator of the institution. Additionally, 4 MRUs also reported the need for obtaining permission from the authorities of concerned departments, generally the head of the departments (HODs). Two MRUs required permissions from Dean of institution, HODs and cost centre (ICU/OT/OPD/IPD) in-charge. In about one-fourth MRUs, additional Institutional Ethics Committee approval was also required. Across all sites, multiple one-on-one meetings were held with the facility head and cost centre in-charge to obtain approval.

### Processes in cost data collection

The process of cost data collection has been summarises highlighting the key issues faced by the different sites in collecting data on input resources (Table 3)

**Table 3:** Overview of cost data collection for different input resources.

Data type		Level of aggregation of data	Source and point of contact	Form of data availability	Common challenges across sites	Potential suggestions from field teams
**Human resource data**						
**HR Salary & Incentives**	Department		1. Accounts officer 2. Accounts clerk 3. Administrative officer	Physical records	Salary information tends to be sensitive, personnel may be hesitant in providing information. Information on allowances and incentives may be difficult to obtain.	Written permission from the head of the institution was observed to be effective in expediting data collection.
						It was also found to be useful to approach central authority instead of individuals to obtain this information.
**Leave**	Department		1. Administrative clerk 2. Administrative officer 3. Head of the department	Physical records	Personnel are usually hesitant to share this information, data is available in physical form at departmental level hence obtaining individual level data is challenging.	Written permission from the head of the institution was observed to be effective in expediting data collection.
						Individual letter and micro-meetings to apprise the staff of the purpose of collecting such data was found to be useful.
**Time allocation**	Individual level		1. Concerned person 2. Proxy interview–Person who has the same routine or knows the routine of the concerned person. 3. Duty roasters	Personnel interview	It required willingness and time of the personnel concerned and is therefore challenging. Secondly there is an issue of over-reporting i.e. people generally tend to over report the time devoted to a particular activity in routine.	Explaining the purpose of this information in costing can improve participation. Scheduling prior appointment or conducting telephonic interviews can save time. For the issue of over-reporting, the investigator summed up time taken for all activities in a day and check whether it matches total working hours. It was also useful to refer to duty roasters for clarity on working hours and rotations.
					Thirdly, it may be challenging to calculate working hours of rotational staff.	
**Physical area/ building**						
**Building area measurement**	Institution		1. Civil engineer 2. Department clerk	Measurement or observation	Records of area may not be available hence physical measurement of area may be required, which can be resource and time intensive.	Most of the hospitals have tiled flooring, counting the number of tiles multiplied with size of tile gives a good estimate of the area.
**Determination of rental price**	Institution		1. Shopkeepers in the same locality 2. Local broker 3. Municipal corporation	Personnel interview	Vast variation in different approaches for determination of rental price.	In case multiple estimates are available, the minimum and maximum rates could be reported.
**Consumables**						
**Consumables used**	Department		1. Staff nurse 2. Store in-charge	Physical records	Data maybe present in disaggregated form in physical register such as a common register for multiple operation theatres. There may be discrepancies in electronic records and actual consumables stock.	If the authorities permit, photographs can be clicked or registers can be photocopied.
**Prices of consumables**	Institution		1. Store head 2. Pharmacist 3. Market survey	Physical records	Data may be received in piecemeal from different sources and may not be available for all items. Physical form of data may make collection time consuming.	It is essential to get data from multiple sources as no one source gives information on all items. It would be useful to have a cost data base from large scale costing studies in Indian settings.
**Non-consumables**						
**Furniture items used**	Department		1. Staff nurse 2. Store head 3. Store Procurement department	Physical records	Records may be available only at institutional or departmental level. There may be discrepancies in records and actual furniture being used.	It is recommended to correlate information in records with physical observation of cost centre and dead stock registers.
**Prices of non-consumable items**	Institution		1. Purchase department 2. Store head 3. Cost centre in-charge	Physical records	Prices of old or donated furniture items may not be available. Additionally these are usually physical records.	It would be useful to have a cost data base from large scale costing studies in Indian settings,.
**Information on average life of furniture items**	Department		1. Cost centre in-charge 2. Store head 3. Clerk	Personnel interview	Information on very old furniture may not be available.	It would be useful to have a standard list from previous costing studies in Indian settings which could act as a repository of data.
**Equipment**						
**Equipment used**	Department		1. Staff nurse 2. Consultant 3. Technician	Physical records	There may be discrepancies between records and physically present equipment.	It is recommended to correlate information in records with physical observation of cost centre.
**Equipment procurement prices**	Department		1. Procurement department 2. Store head 3. Online sources	Physical records	Records may be available only at departmental level. Records of old equipment may not be available.	
**Average life of equipment**	Department		1. Equipment manual 2. Cost centre in-charge 3. Technician	Physical records or Personnel interview	Data might not be readily available, and data obtained tends to be subjective.	Expert opinion may be resorted to for this information, from individuals such as the technician or doctor. The institute may have a condemn policy. This information may be considered for average life.
**Usage of equipment in different procedures**	Department		1. Senior resident 2. Technician 3. Consultant	Personnel interview	This data is not readily available and is contingent upon the availability of personnel.	It is useful to prepare a list of procedures and ask personnel to put a tick against the ones in which the equipment is used.
**Overheads**						
**Electricity**	Institution		1. Engineer 2. Electrician 3. Electricity board	Physical records	Problems may be faced in identifying the right person to obtain information	
**Building Maintenance**	Institution		1. Engineering department 2. Accounts office	Physical records	There may be some hesitation in sharing financial information of the institution.	
**Equipment Maintenance**	Department		1. Store in-charge 2. Clerk	Physical records	There may be some hesitation in sharing financial information of the institution.	
**Laundry**	Institution		1. Laundry in-charge 2. Accounts branch	Physical records	Data maybe available at institution level. The charges may be based on weight and maybe different for each item.	The amount would need to be apportioned to various departments being serviced by the laundry service.
**Dietetics**	Institution		1. Dietician 2. Accounts branch 3. Staff nurse	Physical records	Availability of concerned person may be a challenge.	It may be useful to take prior appointment.
**Biomedical waste management**	Institution		1. BMWM In-charge 2. Accounts branch 3. Officer clerk	Physical records	Data may not be readily available.	Total institutional expenditure and total number of beds may be determined to calculate per bed expense.
**Service provision data**						
**Annual patient load data (OPD/IPD/ Surgeries)**	Specific to procedure		1. Staff nurse 2. Cost centre in-charge	Physical records	The records may not be disaggregated by procedure, number of patients undergoing a certain surgeries would have to be derived from the main records.	
**Average time of each procedure**	Specific to procedure		1. Consultant 2. Senior resident	Personnel interview	Contingent upon identifying the right person and their availability for the interview.	
**Average length of stay in ICU and IPD**	Specific to procedure for surgeries. Specific to departments in case of ICU and IPD.		1. Consultant 2. Senior resident	Personnel interview or physical records	Contingent upon identifying the right person and their availability for the interview.	
					Physical records may be tedious to extract data out of.	
**Diagnostics used in each procedure**	Specific to procedure		1. Consultant 2. Senior resident	Personnel interview	Contingent upon identifying the right person and their availability for the interview.	
**Average OPD visits (pre and post procedure)**	Specific to procedure		1. Consultant 2. Senior resident	Personnel interview	Contingent upon identifying the right person and their availability for the interview.	
**List of drugs and consumables purchased by patient before procedure**	Specific to procedure		1. Consultant 2. Senior resident	Personnel interview	Contingent upon identifying the right person and their availability for the interview.	

Some of the key results are highlighted here:

#### Human resources

Collecting data on human resource time allocation involved personnel interviews. This required willingness and time of the personnel which was challenging. Secondly people generally tend to over report the time devoted to a particular activity in routine. This was evident when the sum total of their activities added to more than their total working hours. This was found in 4 sites and corrections had to be made by re-interviewing the person concerned.

#### Building area & valuation

Determination of building rental price was found to be challenging with wide variation in the estimates from different sources. Different sites followed different approaches to determine the building rental price. The most common sources were market survey and housing rentals in the same area. The final estimate was derived from multiple sources (in 9 of 11 sites), with a preference to commercial rates. All sites relied on physical measurement and blue prints of the building or other records that detailed the area measurement.

#### Equipment & furniture

Determining the average useful life of equipment & furniture items was reported to be difficult due to the absence of records. All sites relied on expert opinion for this estimate. Secondly, cross checking of equipment & furniture lists with physical observation was found to be essential, to determine the functional status of the items.

#### Price data sources

**Fig 3:**
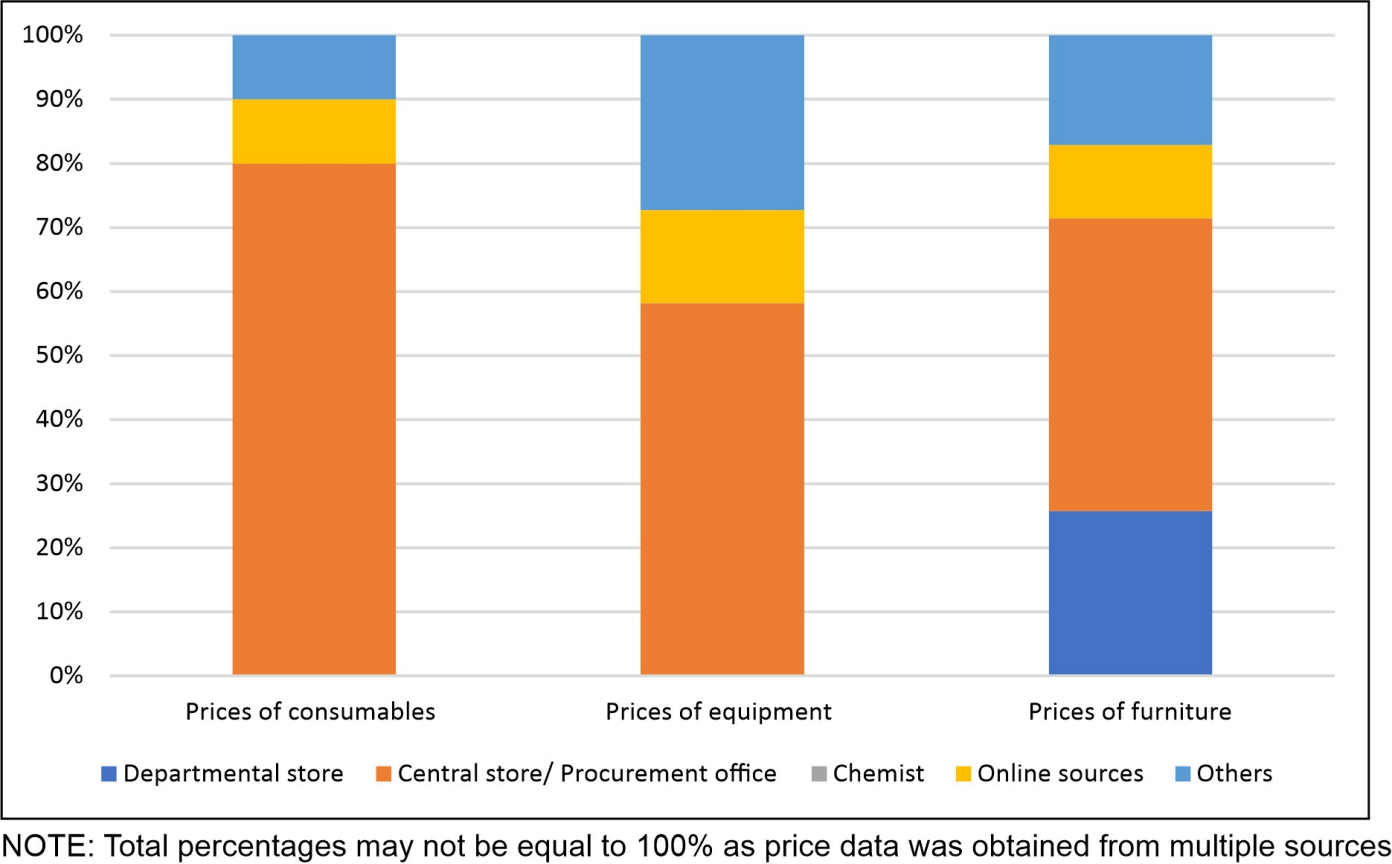
Sources of data for price of consumables, equipment and furniture items.

The price data of consumables, furniture items and equipment was often scattered and multiple sources were required to obtain complete information.(Fig 3)The most common source of consumable and equipment prices was the procurement office or central store, followed by online sources (such as online price databases, procurement portals). Additionally, information from the State Medical Procurement and Supplies department was sought for equipment prices. For furniture items prices, the central and departmental stores were the main sources.

#### Form of data sources

**Fig 4:**
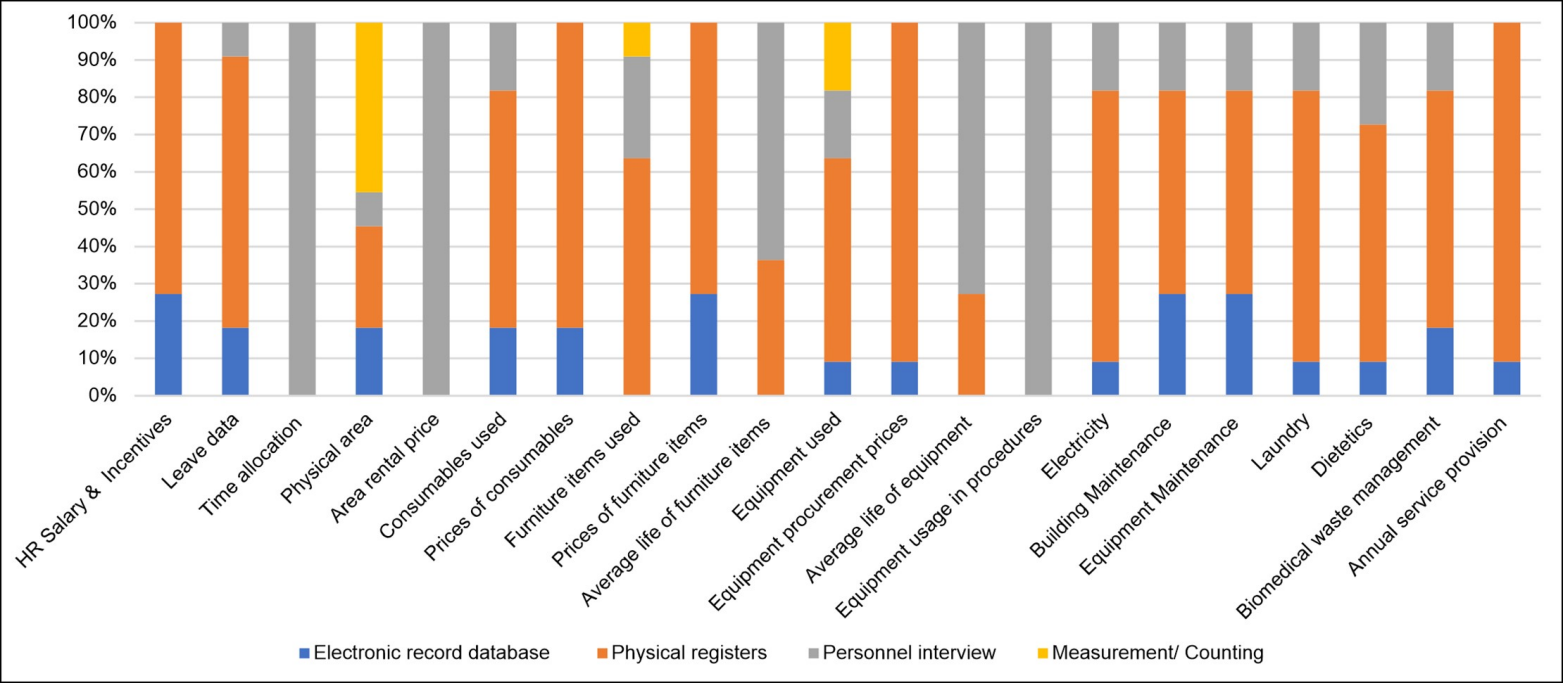
Types of sources of different input resources.

Fig 4 shows the variation in the form of data across input resources. Electronic databases were not available for data collection at most sites. The most common source of data was physical records maintained in respective departments of the hospital. In other cases where the required data was not available, expert opinions were sought.

#### Shared costs

Shared resources often require additional data collection so as to enable apportioning of shared costs. The additional data included the duration of procedures conducted in the same premises collected through staff interview, number of patients sharing common resources collated from hospital records, time schedule of operation theatre collected from the hospital administration.

#### Level of difficulty of data collection

**Fig 5:**
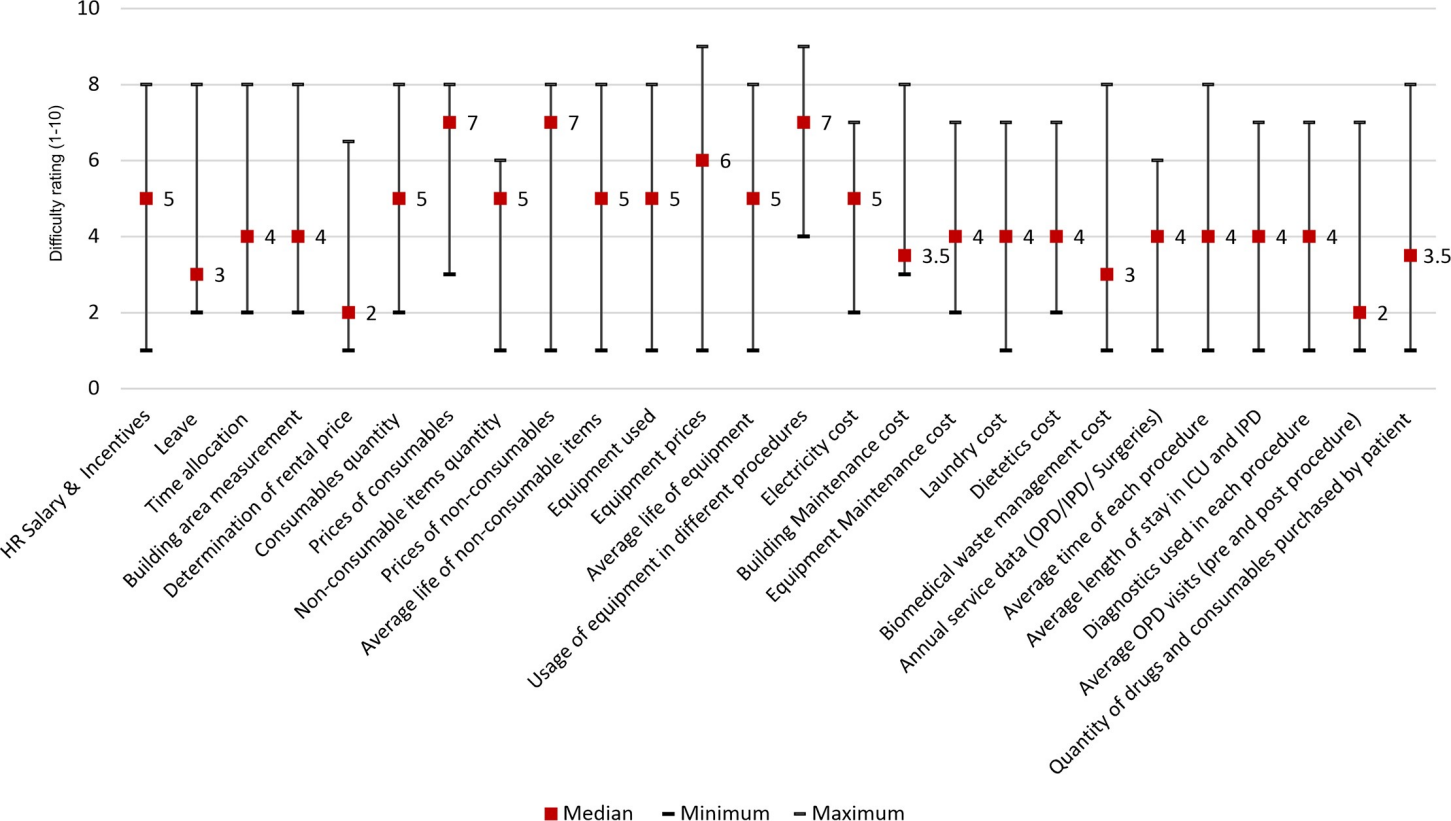
Rating of different resources on difficulty scale (1–10) in cost data collection.

The levels of difficulty in cost data collection as rated by the MRUs are shown in Fig 5. Input resources with high difficulty rating for data collection included enlisting utility of equipment in different procedures (6.59±0.52), prices of consumables (6.09±0.58), equipment procurement price (6.05±0.72) and prices of furniture items (5.64±0.68).

### Results of the group discussion

Key discussion points (Table 4) included determination of rental price and its uniformity across sites, multiplicity of permissions required for data collection, unavailability of electronic hospital records and operational issues such as release of funds, authorization letters from central agency etc.

**Table 4:** Key considerations in group discussion.

DISCUSSION AREA	KEY POINTS
Determination of rental price	• Multiple sources of rental prices used across sites such as government department rental prices, interview of real estate agents, circle rates and expert opinion of local staff. • Wide variation in rental estimates of the same area using different sources. • Lack of one source that may be used across centres.
Multiplicity of permissions required prior to data collection	• Often permission was required to be taken from multiple levels and conducting multiple meetings became essential. • It was noticed that having an approval letter issued from the head of the institution proved useful in decreasing such delays. • Conducting stakeholder meetings with key hospital personnel was found to be useful in smoothening the process and reducing delays.
Unavailability of electronic records	• Hospital records were often available only in physical form. • This increased the data extraction time and also introduced space for inaccuracies which may be in maintenance or extraction of data.
Operational issues	• Multiplicity of organizations involved in the governance of the health system led to the requirement of multiple permissions, which in turn caused operational delays.
Changing data requirements and evolvement of data collection tool	• Revisions in data entry tool multiple times led to delays due to change in output requirements. • Procedural delays due to changes in programmatic requirements from the study.

### Evaluation from field teams on training, supervision and quality assurance

#### Training for cost data collection

Hands on training and on-site support from the Central Supervisory team after the initial training was found to be very useful. Key concepts requiring further discussion and training were reported to be time allocation, medical terminologies, cost data collection in specific cost centres such as operation theatres. Participants suggested increase in time spent on practicum during the training and more frequent(quarterly) trainings and workshops.

Supervisory supports: Supervisory support by the central team and co-investigators who were trained at the central level was found to be of paramount importance in addressing day to day queries and conceptual understanding. It was suggested that the central team visit the sites to overlook data collection and provide hand-holding, especially with regards to data collection on revenue, time allocation and shared costs.

#### Quality assurance

Quality assurance was of great importance, in particular to ensure standardisation and comparability in this multi-centre study. Key quality assurance strategies were highlighted in stakeholder interviews stating four levels of quality checks, namely checks during data collection, data entry, data sharing and finally on receipt of the data. During data collection, correctness and appropriateness of data was checked, for instance cross checking of time allocation hours. At the time of data entry correctness of entry was checked. Before sharing with the central team, the data was verified by the field supervisor. Finally the central team which carried out the analysis identified data gaps and discrepancies which were then communicated to the field teams. Teams then addressed those gaps by clarifying doubts, recollection of required data and re-entry of any incorrect data.

## Discussion

Costing require information on numerous input resources and each of which have distinct data collection methods, making such activities rigorous and time consuming. Additionally, factors such as nature of costing (prospective or retrospective), costing methodology (top down or bottom up), form of data, number of people involved and willingness to share data, define the kind of challenges and time delays that may be faced during data collection. [30] Operational and methodological issues in cost data collection are further enhanced in developing countries like India with increased time taken to collate, input and assure data quality due to limited data availability, multiplicity of sources and unavailability of digitized data. [28,29] This study was targeted at identifying factors and barriers affecting cost data collection and potential solutions, in Indian and similar resource settings.

The study found that the average time required for cost data collection at one tertiary level public facility was 7 months or 355 person-days, inclusive of time required for permissions, actual data collection and data entry, queries & final submission, inclusive of delays such as non-working days. This was found to be higher than some other Indian studies which reported 25 and 45 days, however they had larger data collection teams with 8 and 20 members respectively. [30]

Our study found that a majority of the time was spent on data verification and clarifications to address erroneous data and address new data requirements. This was found to be consistent with other studies which reported that data verification could take longer than planned because incomplete or incorrect data. [30] A thorough pilot test of the tool to ensure its appropriateness and understanding of the data requirement by data collection team is the first step to improve the data quality and avoid erroneous data. Training was found to be essential in building an understanding about basics of costing, data requirements and importance of the costing activity amongst the data collection teams. Practical exposure to cost data collection was especially reported to be useful. The model of Training of Trainers at central level and subsequent training of data collectors by master trainers at local level was found to be useful by the trainees. The continued support from the central level team was also critical in local teams’ activities and the local teams stated the need to have further support in the form of on-site visits. Time was also lost due to the multiple levels of permissions required, gaining full participation of facilities and logistical delays. The permission requirements varied across the settings, however having an official consent letter from the head of the institution, holding stakeholder meetings and assuring confidentiality helped to gain confidence and smoothen the data collection process, ultimately reducing delays. This is keeping with methods employed in other studies. [30]

### Implications for future research

The study identified certain areas of data collection that were more challenging than others and might require additional inputs or careful advance planning. The key lessons learned from this are summarised in (Table 5). An order of priority in data collection has been assigned to different data based on their share in total cost, difficulty level and level of uncertainty. The data collectors rated the prices of consumables, furniture, equipment and utility of equipment in different procedures as most difficult data type to collect. Determination of accurate building rental price was also difficult as there was no single source for this data. Prices and average useful life of the old furniture and equipment were not available as back dated records were not maintained at most facilities.

**Table 5:** Summary findings and implications for cost data collection.

Cost head	Ranking in order of share in cost (% share of cost)	Data type	Median rating of Difficulty of collection (Scale of 1–10)	Level of uncertainty in variable *	Prioritization in data collection*	Possible type of data collector (T/NT) **
**Human resource**	**1 (41%)**	HR Salary & Incentives	5	**+**	**+++**	**NT**
		Time allocation	4	**+++**	**+++**	**T**
		Leave	3	**++**	**++**	**NT**
**Consumable items**	**2 (36%)**	Prices of consumables	7	**+++**	**+++**	**NT**
		Consumables used	5	**+**	**++**	**T**
**Physical area/ building**	**3 (11%)**	Determination of rental price	2	**+++**	**+++**	**NT**
		Building area measurement	4	**+**	**++**	**NT**
**Equipment**	**4 (5%)**	Equipment used	5	**+**	**++**	**T**
		Equipment prices	6	**+++**	**++**	**NT**
		Average life of equipment	5	**+++**	**++**	**T**
		Usage of equipment in different procedures	7	**++**	**++**	**T**
**Utility**	**5 (3%)**	Dietetics	4	**+**	**+**	**NT**
		Laundry	4	**+**	**+**	**NT**
**Overheads**	**6 (2%)**	Electricity	5	**+**	**+**	**NT**
		Building Maintenance	3.5	**+**	**+**	**NT**
		Equipment Maintenance	4	**+**	**+**	**NT**
		Biomedical waste management	3	**+**	**+**	**NT**
**Furniture / Non-consumable items**	**7 (1%)**	Furniture items used	5	**+**	**+**	**NT**
		Prices of non-consumable items	7	**+++**	**+**	**NT**
		Average life of furniture items	5	**+++**	**+**	**T**

* +: Low; ++: Moderate; +++: High

** T(Technical): Personnel with experience in costing cost data collection; NT(Non-technical): Personnel with understanding of the health system functioning

At the onset of cost data collection, decisions need to be made regarding key input resources to be prioritised, key data sources, time and human resources required for each set of data. As the same assumptions can be followed across centres in multi-centre costing studies, a reference data base for prices and average useful life of equipment and furniture items would save time. A national health system cost database which has been developed for India does make an attempt to bridge this limitation by providing representative data on prices and salaries. [31] This type of streamlining would be a cost-effective solution and is particularly justifiable in the case of equipment which only contributed 5% of the total cost.

Human resource data needs to be collected and handled carefully, since it has a major impact on the total cost. The combined cost of human resources and drugs & consumables accounted for 41% of the total cost, however, it required only 19% of total data collection time. It is essential to carefully tally time allocation tables of all human resources, delineate activities of the personnel and check over-reporting of service provision time. A barrier common to data collection of all resources was data extraction from physical records and registers, which is cumbersome and time-consuming. Sites with insufficient information management system have been known to have a high effort of conducting cost data collection which may even make it non-feasible for the government. [32] Availability of reliable electronic health records would facilitate ease of data collection and prevent time delays and is likely to have beneficial effects in other areas such as patient care and facility management. In addition, it is important to use the skill mix of interviewers for appropriate nature of data collector. Data on items such as overheads, biomedical waste, leave record, consumable indent, building area measurement generally require less expertise and may be collected by non-technical staff. On the other hand, time allocation of human resources and utility for various resources, which are used for apportioning, requires an understanding of the systems and processes and hence need more technical staff for its collection.

**Fig 6:**
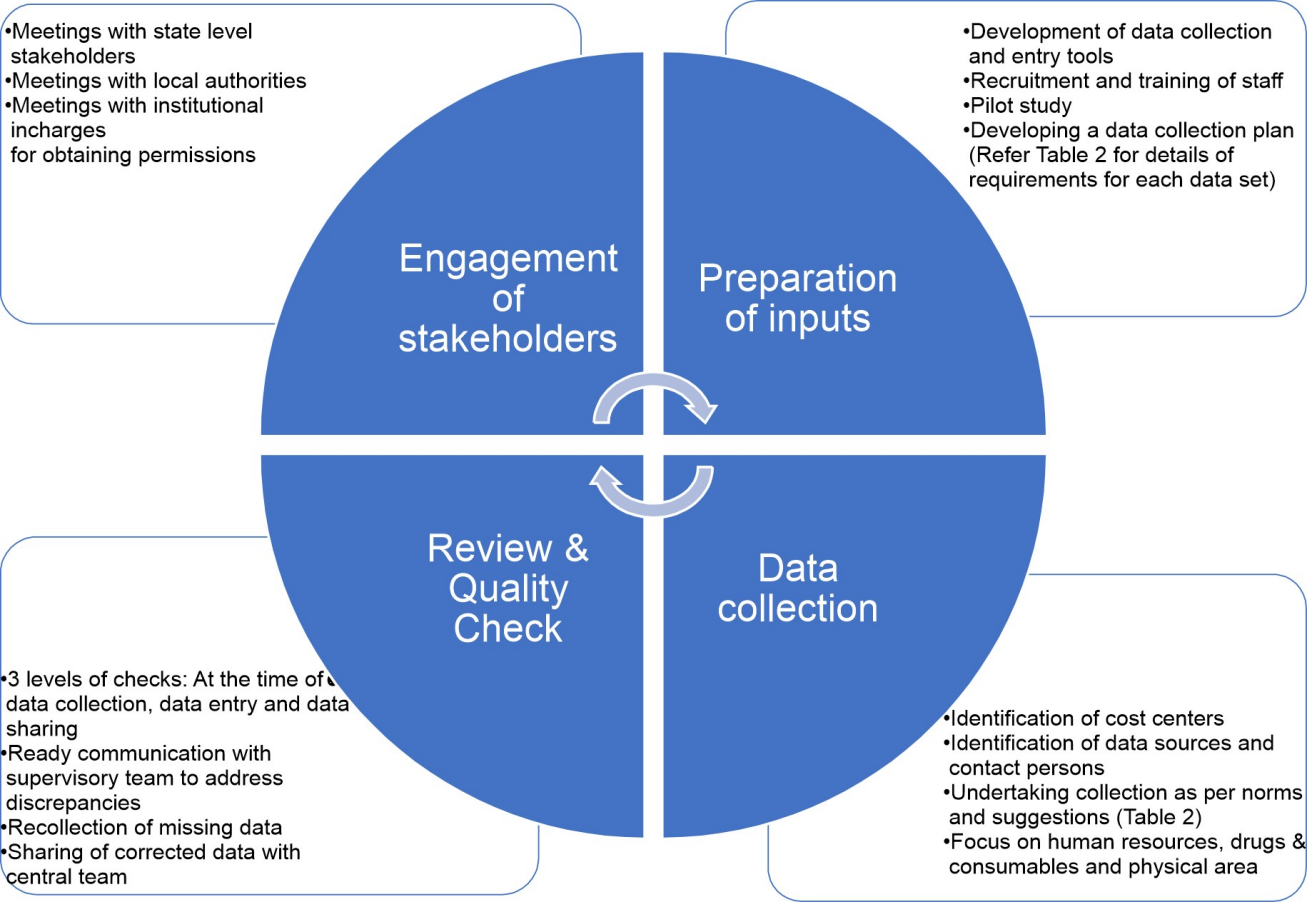
Framework for planning cost data collection.

Our study findings suggest that during planning of cost data collection, it is essential to remember four important sets of considerations. (Fig 6) The first step is to engage all stakeholders to develop an understanding about the significance of the costing activity, type of data required and streamlining permissions which helps in prevention of delays during data collection. Engagement with stakeholders also builds ownership in the findings for the study. This is particularly relevant if the costing is being done for the purpose of price setting or reimbursement. Next is preparation of all inputs for cost data collection including training, mentoring support and conducting a pilot study. Thirdly, development of a data collection plan specifying cost centres, data sources, norms and suggestions for best practice approaches (Table 3). The final consideration is review and quality check of the data. Based on the CHSI experience, there should be a system of checks in place at four levels: at the time of data collection, data entry, data sharing and finally on receipt of the data. At all steps of the data collection process, it is important to keep a communication system between the site team and the supervisory team, and between the supervisory team and other stakeholders.

The lessons learned are particularly relevant for two sets of reasons. Firstly, the HTA studies are being commissioned in large numbers which require cost evidence to begin with. Secondly, the emergence of publicly financed health insurance schemes such as AB–PMJAY require evidence on cost to set prices for provides. As a result of these twin developments, it is expected that there will be a rise in number of cost analysis studies. Hence, our study findings are likely to be useful in design, conduct and quality assurance of those future studies. While the findings of this study are based only on public tertiary level care, they can be used to infer about any cost assessment to be undertaken at primary and secondary care levels as well. Although there are likely to be differences in the data sources and time required for data collection, the main principles of collecting information on different resources, key assumptions and barriers are likely to be similar.

We would like to note certain study limitations. Firstly, while we assess the level of difficulty faced in data collection, we could not assess and report in quantitative terms. Moreover, this quality assessment, if done according to skill-mix of data collection can provide very useful insight into which type of data collection could be assigned which type of data collection. Further, the difficulty rating was based on a joint decision of the data collection team and not an individual, hence it is not possible to comment on the impact of skill mix of the data collector on data quality. Hence, our current recommendation is based more on the level of difficulty faced and importance of particular data in extent and accuracy of cost, rather than quality of data collected. Secondly, while we collected the time spent in person-days on different tasks for each individual health facility and cost centre, this data was not available disaggregated by individual staff with different level or skill mix. As a result, accurate estimation of the cost of data collection for individual activities or individual cost centres was not possible. Thirdly, this paper focussed on findings from tertiary hospitals where costing was under taken as a part of the CHSI study. Similar studies may be done in the future for district hospitals, private hospitals and other healthcare delivery levels where the CHSI study is being undertaken. Thirdly, there is a significant likelihood of differences in the processes and systems for data collection in private sector hospitals, and more decentralized public sector facilities such as district hospitals, CHCs and PHCs. In view of the large contribution of private sector in service delivery in India, understanding the process of costing in these facilities becomes imperative. Finally, we have highlighted the issues in the context of a study which follows a mixed methodology of top-down and bottom-up. It will be useful to replicate this study in the context of pure bottom-up and top-down costing methodologies, as well as studies from a financial and economic perspective, so as to draw specific lessons for each of the different methods.

## Conclusion

The Government of India is committed to health technology assessment as it is the need of the hour. Improving methodology and practical implementation of costing studies will improve the quality of estimates being generated and ultimately aid national priority setting. For implementation of costing studies, having clearly outlined outputs, conducting a pilot test, having a quick communication between the data collectors and central team, specific quality checks and assumptions, are key considerations. In addition to practical aspects, it is essential to strengthen the health system to aid costing studies. Developing electronic health records, having nationally representative cost data databases for reference in case of unavailability of data and developing guidelines on costing in Indian settings are key deliberations.

## Supporting information

S1 Data Online survey tool. (DOCX)

S2 Data Online survey data. (XLSX)
